# Data on nitric oxide production by human bone marrow-derived mesenchymal stromal cells

**DOI:** 10.1016/j.dib.2016.07.021

**Published:** 2016-07-19

**Authors:** Mehdi Najar, Mohammad Fayyad-Kazan, Hussein Fayyad-Kazan, Nathalie Meuleman, Dominique Bron, Laurence Lagneaux

**Affiliations:** a Laboratory of Clinical Cell Therapy, Institut Jules Bordet, Université Libre de Bruxelles (ULB), Campus Erasme, Brussels, Belgium; b Institut de Biologie et de Médecine Moléculaires, Université Libre de Bruxelles, 6041 Gosselies, Belgium; c Laboratory of Cancer Biology and Molecular Immunology, Faculty of Sciences I, Lebanese University, Hadath, Lebanon

## Abstract

Due to its anti-inflammatory and immunosuppressive potential, Nitric oxide (NO), a gaseous radical, is of special importance during graft-versus-host diseases (GVHD) and feoto-maternal tolerance. NO is a major mediator of murine mesenchymal stromal cells (MSCs)-immunosuppressive capacity. In this data article, we characterized NO production by human bone marrow-derived MSCs (hBMSCs). MSCs, isolated from healthy donors (*n*=5), were defined according to the International Society for cellular Therapy (ISCT) guidelines. Based on a fluorometric detection system, and upon using Nitrite (${NO}_{2}^{-}$)/Nitrate ( ${NO}_{3}^{-}$) Assay Kit, the amounts of NO metabolites ( ${NO}_{2}^{-}$ and ${NO}_{3}^{-}$) produced by hBMSCs, being grown in a culture medium either lacking (constitutive condition) or containing IL-4, IL-10 or a pro-inflammatory cytokine cocktail made of IL-1β, TNF-α, IFN-α and IFN-γ, were assessed. All assays were carried out in triplicates and the mean values are reported. The data from this study supports and corroborates the discussion associated with our previously published work entitled “The Immunomodulatory Potential of Mesenchymal Stromal Cells: A Story of a Regulatory Network” (Najar et al., 2016) [1].

**Specifications Table**

**Table:** 

Subject area	*Biology*
More specific subject area	Mesenchymal stromal cells (MSCs)
Type of data	Figure
How data was acquired	Fluorometric detection of nitric oxide metabolites
Data format	Analyzed
Experimental factors	hBMSCs were cultured under basic state or treated with IL-4 (50 ng/ml), IL-10 (50 ng/ml) or a pro-inflammatory cytokine cocktail containing IL-1β (25 ng/ml), TNF-α (50 ng/ml), IFN-α (10 ng/ml) and IFN-γ (50 ng/ml)
Experimental features	NO^−^_2_ and NO^−^_3_ production levels by hBMSCs were assessed using the Nitrite/Nitrate Assay Kit (Sigma)
Data source location	Institut Jules Bordet, Brussels, Belgium
Data accessibility	Data are provided in the paper

**Value of the data**

• This data provides evidences that NO is not constitutively produced by hBMSCs
• This data will be beneficial for the scientific community focusing on understanding the molecular mechanisms by which hBMSCs modulate the function of their target cells
• This data could help to understand the varied therapeutic efficiency exhibited by MSCs-derived from different organisms.

## 1 Data

**Fig. 1:**
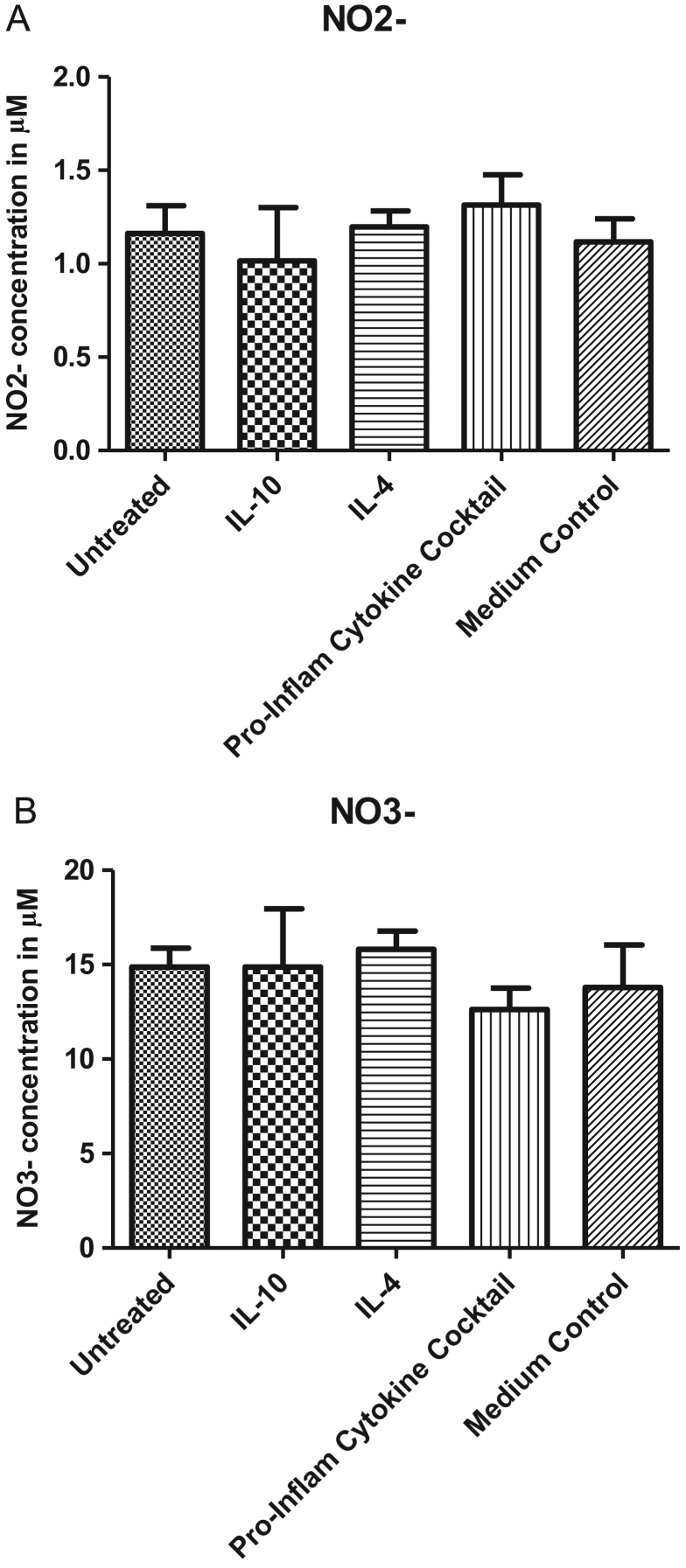
NO metabolite production by hBMSCs. hBMSCs were cultivated under both basic and cytokine primed conditions. Cell priming was performed by treating cells (overnight) with IL-10, IL-4 or a pro-inflammatory cytokine cocktail (IL-1β, TNF-α, IFN-α and IFN-γ). The supernatant of hBMSCs was collected and NO concentration was measured by determining ${NO}_{2}^{-}$ and ${NO}_{3}^{-}$ levels upon following the guidelines provided by the Nitrite/Nitrate Assay Kit (Sigma). Values reported represent the Mean concentration (µM) of ${NO}_{2}^{-}$ (*Panel A*) and ${NO}_{3}^{-}$ (*Panel B*)±SD. Fresh culture medium lacking cells was used as a blank and served as a control.

The data presented here show Nitrite ( ${NO}_{2}^{-}$) (Fig. 1, Panel A) and Nitrate ( ${NO}_{3}^{-}$) (Fig. 1, Panel B) concentrations detected in the culture media after cultivating hBMSCs in the absence or presence of IL-4, IL-10 or a pro-inflammatory cytokine cocktail containing IL-1β, TNF-α, IFN-α and IFN-γ.

## 2 Experimental design, materials and methods

### 2.1 Isolation and cultivation of hBMSCs

This study was conducted in accordance with the Declaration of Helsinki (1964) and after approval of the ethics committee of the “Institut Jules Bordet” (Belgium). BM was obtained either from the sternum or iliac crest of healthy donors (*n*=5). Informed written consent is obtained from each donor. Briefly, mononuclear cells (MNCs) were isolated from bone barrow aspirates by density-gradient centrifugation (LinfoSep, Biomedics, Madrid, Spain) and then washed in Hanks׳ Balanced Salt Solution (HBSS, Lonza Europe, Verviers, Belgium). MNCs were seeded at 2×10^4^ cells/cm^2^ in low glucose Dulbecco׳s Modified Eagle׳s Medium (DMEM-LG, Lonza) supplemented with 15% fetal bovine serum (FBS, Sigma-Aldrich, Bornem, Belgium), 2 mM L-glutamine and 50 U/ml penicillin (both from Lonza). Cells were then incubated at 37 °C in a 5% CO2-enriched humidified atmosphere, cultured up to 80–90% confluency, trypsinized (Lonza), centrifuged, and expanded by subculturing at a lower density (1000 cells/cm2). MSCs were analyzed under both constitutive (control) and cytokine primed conditions. Cell priming was carried out by treating cells (overnight) with IL-10 (50 ng/ml), IL-4 (50 ng/ml) or a pro-inflammatory cytokine cocktail containing IL-1β (Peprotech, Rocky Hill, NJ, USA) (25 ng/ml), TNF-α (50 ng/ml), IFN-α (3000 U/ml or 10 ng/ml) and IFN-γ (1000 U/ml or 50 ng/ml) (all from Prospec Inc., Rehovot, Israel).

### 2.2 NO detection

After culturing hBMSCs under the different conditions (basic *versus* cytokine primed), their supernatants were collected by centrifugation where Nitrite (${NO}_{2}^{-}$) and Nitrate (${NO}_{3}^{-}$ ) concentrations were determined using the Nitrite/Nitrate Assay Kit (Sigma). Using this assay, fluorometric detection of Nitrite (${NO}_{2}^{-}$) concentration, in a sample, was achieved upon allowing a chemical reaction between Nitrite (${NO}_{2}^{-}$) and 2,3-diaminonaphthalene (DAN) to generate a fluorescent product called naphthotriazole. The fluorescence intensity was read using a fluorescence reader (FLUOstar Optima; BMG Labtech, Ortenberg, Germany) with excitation/emission wave lengths of 360 nm/450 nm. For determination of Nitrate (${NO}_{3}^{-}$) concentration in a sample, ${NO}_{3}^{-}$ was first converted to ${NO}_{2}^{-}$ by the enzyme Nitrate Reductase, where the measured fluorescence-intensity corresponds to the total [${NO}_{2}^{-}$+${NO}_{3}^{-}$] amount in the tested sample. Then, the previously measured [${NO}_{2}^{-}$] concentration was subtracted from the obtained [${NO}_{2}^{-}$+${NO}_{3}^{-}$] value. Accordingly, the Nitrate (${NO}_{3}^{-}$) concentration was calculated according to the formula: [${NO}_{3}^{-}$ ]=[${NO}_{2}^{-}$+${NO}_{3}^{-}$ ]−[${NO}_{2}^{-}$ ]. Noteworthy that, [${NO}_{2}^{-}$] and [${NO}_{2}^{-}$+ ${NO}_{3}^{-}$] values were determined using standard curves.

### 2.3 Statistical analysis

The data were analyzed with Optima software (BMG Labtech, Ortenberg, Germany). Three separate experiments were carried out in triplicate and averaged. Data are presented as means ± SD and analyzed using Wilcoxon Signed Rank test. *P*-Values<0.05 were considered significant.
