# Supplementary material for: Data on nitric oxide production by human bone marrow-derived mesenchymal stromal cells
Source: Data Brief. 2016 Jul 19;8:1111–4. doi: 10.1016/j.dib.2016.07.021 (PMC4976668; doi:10.1016/j.dib.2016.07.021)
Supplement: Supplementary file 1 — Supplementary material [file mmc1.docx]

Conflicts of interest

We wish to confirm that there are no known conflicts of interest associated with this publication.
